# The Clinical Importance of Differentiating Monogenic Familial Hypercholesterolemia from Polygenic Hypercholesterolemia

**DOI:** 10.1007/s11886-022-01783-5

**Published:** 2022-09-09

**Authors:** Fistra Janrio Tandirerung

**Affiliations:** The Institute of Cardiovascular Science, University College London (UCL), Gower Street, London, WC1E 6BT UK

**Keywords:** Low-density-lipoprotein cholesterol (LDLC), Familial hypercholesterolemia, Monogenic, Polygenic hypercholesterolemia

## Abstract

**Purpose of Review:**

The current review discusses the importance and significance of differentiating monogenic familial hypercholesterolemia (FH) from polygenic hypercholesterolemia for clinical purpose.

**Recent Findings:**

Consistent scientific evidence have demonstrated that, compared to polygenic hypercholesterolemia, monogenic FH patients are at significantly higher risk for premature coronary heart disease (CHD). This is despite both disease entities having a comparable low-density-lipoprotein cholesterol (LDLC) level. Monogenic FH also has poorer therapeutic response compared to its polygenic counterpart. However, there are no current available clinical management guidelines that stratify hypercholesterolemia patients based on genotype.

**Summary:**

Monogenic FH patients are at higher risk for CHD with poorer therapeutic response. Thus, genotype testing should be performed when available. There is also an urgency to develop genotype-based clinical guideline that stratify patients on genotype and not only based on traditionally known cardiovascular risk factors.

## Introduction

Familial hypercholesterolemia (FH) is a disease characterized by a severe increase of low-density lipoprotein cholesterol (LDLC) due to genetic mutations. The prevalence is generally around 1:250–500 individuals [1, 2•]. FH presents a marked increase of LDLC, mainly due to mutations in the low-density lipoprotein receptor (*LDLR*) gene. A small proportion of FH is caused by apolipoprotein-B (*APOB*) and proprotein convertase substisilin/kexin type 9 (*PCSK9*) mutations [2•]. All those mutations result in impaired LDLC clearance from circulation, leading to a severe LDLC increase. Furthermore, the severe increase of LDLC that occurs from the early years of life is associated with premature episodes of coronary heart disease (CHD) [3].

However, mutations of the aforementioned genes are identified in only about 40–60% of clinically diagnosed FH cases [1, 4•, 5••]. The condition in which no single genetic mutation (monogenic) can explain the severe hypercholesterolemia is likely to be polygenic hypercholesterolemia. The marked LDLC increase in polygenic hypercholesterolemia is due to the cumulative effect of multiple LDLC-raising single nucleotide polymorphisms (SNPs) in a same individual [2•].

Several studies have demonstrated that monogenic FH and polygenic hypercholesterolemia can have different lipid and disease presentations as well as responsiveness to lipid-lowering therapy [4•, 5••, 6]. However, the clinical significance of differentiating monogenic FH from polygenic hypercholesterolemia remains unclear [4•]. The necessity to formulate different treatment approaches for different genotypes is also poorly investigated [2•]. Therefore, this essay aims to discuss the difference between monogenic FH and polygenic hypercholesterolemia in terms of cardiovascular risk profile and response to lipid-lowering treatments, and how this might affect clinical management.

## Cholesterol Metabolism Overview

Approximately four-fifth of total body cholesterol is internally synthesized (mainly in hepatocytes), while the remaining one-fifth is derived from diet [7]. In the hepatocyte cytoplasm, 3-hydroxy-3-methylglutaryl(HMG)-CoA synthase (HMGCS) synthesizes HMG-CoA from acetyl-CoA. The further processes occur in endoplasmic reticulum (ER). HMG-CoA is reduced by HMG-CoA reductase (HMGCR) into mevalonate (mevalonic acid). Mevalonate, through the sequence of enzymatic processes, is metabolized to form cholesterol as the final product [8].

In liver cells, cholesterol is packed into very low-density lipoprotein (VLDL) molecules that contain several apolipoproteins, including Apo-B. VLDL is released into circulation and transformed into low-density lipoprotein (LDL) by lipoprotein lipase (LPL) [7, 9]. Upon transformation into LDL, Apo-B is the only remaining apolipoprotein in LDL molecules. Apo-B acts as a ligand for LDLR on target cells (primarily hepatocytes), hence facilitating the LDL-LDLR binding process [9, 10]. The LDL-LDLR complex is further internalized into the target cells and dissociates, releasing free cholesterol while LDLR is recycled back to the cell surface [11].

The LDLRs will be internalized and undergo lysosomal degradation in hepatocytes. This process is mediated by PCSK9 that binds to LDLR at the cell surface. The LDLR-PCSK9 complex is further internalized and degraded in the lysosome, resulting in reduced LDLR amount and therefore reduced LDLC uptake [7]. This LDLR degradation is regulated based on ER cholesterol concentration. High ER cholesterol concentration increases LDLR degradation, which prevents further LDLC uptake and vice versa [12].

## Monogenic Familial Hypercholesterolemia

Monogenic FH is caused by mutations mainly in the *LDLR*, *APOB*, and *PCSK9* genes. These mutations are autosomal dominant (one inherited mutated allele can cause FH). Most monogenic FHs (approximately 1:250–500 in prevalence) are heterozygous (mutations affect only one allele) [10]. The LDLR gene mutations account for about 80% of total monogenic FH cases, while Apo-B and PCSK9 mutations account for 5% and < 1%, respectively [10, 13]. A small proportion (up to 1:1,000,000 in prevalence) of FH is homozygous in which both alleles (mainly *LDLR*) have the same mutation, therefore exhibits more severe lipid and clinical phenotypes secondary to severe LDLR functional loss [9, 14].

*LDLR* mutations leading to monogenic FH are loss-of-function mutations. These mutations can cover a broad spectrum of LDLC level based on the functionality of LDLR [14]. Hence, LDLR activity in homozygous FH is categorized as receptor-negative (≤ 2% LDLR activity) and receptor-defective (2–25% LDLR activity) [3]. Heterozygous FH can retain up to 70% of total LDLR activity [14]. LDLR activity is inversely related to plasma LDLC level. Therefore, receptor-negative patients (also known as null variant) experience the most severe phenotype (highest LDLC), while receptor-defective patients exhibit lower LDLC level depending on LDLR functional reserve [14].

APOB gene mutations that lead to FH are also loss-of-function mutations, resulting in defective LDLC binding to LDLR. Contrary to *LDLR* and *APOB* mutations, in FH, *PCSK9* mutations are gain-of-function mutations. This leads to increased LDLR degradation in the lysosome and decreased LDLR availability for LDLC uptake [11].

Phenotype presentations of heterozygous FH are much more affected by external factors such as age, diet, lifestyle, or medications. Inversely, homozygous FH phenotypes are usually independent of the other risk factors [3]. Therefore, addressing other risk factors can have additional benefit for heterozygous FH patients. Homozygous FH can reach a total cholesterol level of above 1000 mg/dl, and those who are untreated may have LDLC of > 500 mg/dl. On the contrary, heterozygous monogenic FH total cholesterol level ranges from 310 to 580 mg/dl with LDLC average is 220 mg/dl in untreated population [9]. This different lipid profile corresponds to the onset of CHD. Untreated homozygous FH patients may die from CHD before the age of 20, while in the heterozygous population is usually after the fourth decade of life [5••, 9]. The further discussion puts emphasis on the heterozygous FH.

## Polygenic Hypercholesterolemia

Polygenic hypercholesterolemia can occur when known mutations (*LDLR*, *APOB*, or *PCSK9* mutations) are not identified in patients clinically diagnosed with FH. Some other rare variants have been shown to be FH-causing variants, such as mutations in *CYP27A1* (cytochrome P450), *LIPA* (lysosomal lipase A), *LIPC* (hepatic lipase), or *LIPG* (endothelial lipase) genes. However, some efforts to identify FH-causing mutations have failed to discover novel mutations [14].

In the absence of monogenic mutations, the involvement of multiple genes is postulated to be the cause, thus referred to as polygenic hypercholesterolemia. Patients with polygenic hypercholesterolemia are carriers of multiple common variants/SNPs that cause LDLC increase [14]. Polygenic hypercholesterolemia is confirmed by polygenic risk score (PRS) that calculates the aggregate of LDL-raising effect of the common variants [15]. Polygenic hypercholesterolemia was first explained by Talmud et al. in which the high LDLC level was found to be due to the cumulative effect of 12 common variants that increase LDLC in circulation. This combined LDL-raising effect, to some extent, can be comparable with the LDLC level in monogenic FH [16]. Over 100 loci influencing LDLC level have been identified [17]. A study demonstrated that monogenic FH had a higher mean LDLC baseline than polygenic hypercholesterolemia (7.6 mmol/l (293.9 mg/dl) vs. 6.2 mmol/l (239.75 mg/dl)) [4•]. In the case of very high LDLC without an identified monogenic mutation, it is likely to a have greater number of LDLC-raising SNPs compared to the average polygenic hypercholesterolemia [18].

However, the polygenic concept can be complicated in terms of disease presentation. The more LDLC-raising SNPs involved, the more severe phenotypes can be observed. Inversely, genes that contribute to LDLC reduction may also be involved, therefore compensate the LDLC-raising effect of other genes and decrease the severity of hypercholesterolemia [5••, 16]. Even more complicated, polygenic involvement can contribute to increasing or decreasing LDLC level in patients with identified monogenic mutations [16]. However, it is essential to underline that unidentified mutations may not necessarily be polygenic but can be due to monogenic mutations with corresponding genes that have yet to be identified, especially when the PRS is low [5••, 19]. Nevertheless, it is expected that approximately 80% of individuals clinically diagnosed with no LDLR, APOB, and PCSK9 mutation identified are likely polygenic hypercholesterolemia [5••].

## Familial Hypercholesterolemia Diagnosis

FH diagnosis mainly relies on physical examination findings, blood cholesterol level, and family history of CHD. Genetic testing, despite being a gold standard for mutation identification, is still debatable for routine clinical practice. Apart from cost and availability issues, some argue that knowing the exact mutation is not essential since the LDLC level is the primary information to guide diagnosis and therapy [18, 20]. However, some others argue that genetic testing should be performed when resources for testing are available. The reason is that, in some mutations, the risk for cardiovascular events is higher, has poorer prognosis, and may require more aggressive treatment [10]. For instance, *APOB* mutations tend to be more responsive to statins than *LDLR* mutations [11], different type of *LDLR* mutations respond differently to statin therapy [21–23], and *LDLR* mutations tend to be more severe in phenotypes than *APOB* and *PCSK9* mutations [24]. Hence, clinical diagnosis is unable to explain the association between genotype and phenotype [11]. Therefore, there are convincing evidences to argue that LDLC-directed therapy alone is not sufficient and patients’ genotype should be considered in providing comprehensive hypercholesterolemia management.

There are 3 main diagnosis criteria that are often used in diagnosing FH, i.e., Simon Broome Register Group (SBRG) (Table 1), Dutch Lipid Clinic Network (DCLN) (Table 2), and Make Early Diagnosis to Prevent Early Death (MedPed) **(**Table 3) [18, 25]. Despite that DLCN and SBRG include genetic testing, there are still no formal therapy guidelines based on different genotypes. Hence, genetic testing is primarily used to determine the definitive diagnosis of FH (DLCN needs additional criteria in addition to genetic mutation) instead of disease stratification and therapeutic approach. The International Atherosclerosis Society stratifies FH patients as severe and non-severe [20]. However, this stratification is based on LDLC and other known atherosclerotic cardiovascular disease risk factors instead of genotype.

**Table 1:** Simon Broom Register Group (SBRG) Criteria [18]

**Criteria**	**Description**
**a**	Total cholesterol (TC) concentration > 7.5 mmol/l (290 mg/dl) in adults, or TC > 6.7 mmol/l (260 mg/dl) in children less than 16 years, or LDLC concentration > 4.9 mmol/l (190 mg/dl) in adults or > 4.0 mmol/l (155 mg/dl) in children
**b**	Tendon xanthomata in the patient or first-degree relatives
**c**	*LDLR* or *APOB* gene mutation
**d**	Family history of myocardial infarction before age 50 years in second-degree relatives or before 60 years in a first-degree relative
**e**	Family history of raised total cholesterol concentration > 7.5 mmol/l (290 mg/dl) in a first-/second-degree relative, or greater than 6.7 mmol/l (260 mg/dl) in child or sibling aged younger than 16 years
Definite FH if - a **and** b, **or** - c	Probable FH if - **a** and **d**, or - **a** and **e**

**Table 2:** Dutch Lipid Clinic Network Criteria [18]

**Criteria**	**Points**
**Family history** - First-degree relative with known premature coronary and vascular disease (men < 55 years; women < 60 years), or - First-degree relative with known LDLC > 95th percentile - First-degree relative with tendinous xanthoma and/or arcus cornealis, or - Children < 18 years with LDLC > 95th percentile	1 1 1 2
**Clinical history** - Patient with premature CHD (men < 55 years; women < 60 years) - Patient with premature cerebral or peripheral vascular disease (men < 55 years; women < 60 years)	2 1
**Physical examination** - Tendinous xanthomata - Arcus cornea < 45 years	6 4
**Cholesterol level** - LDLC ≥ 8.5 mmol/l (≥ 329 mg/dl) - LDLC 6.5–8.4 mmol/l (251–329 mg/dl) - LDLC 5.0–6.4 mmol/l (193–251 mg/dl) - LDLC 4.0–4.9 mmol/l (155–193 mg/dl)	8 5 3 1
**Functional *LDLR* gene mutation**	8
**Diagnosis** - Definite FH if > 8; probable FH if 6–8; possible FH if 3–5

**Table 3:** Make Early Diagnosis to Prevent Early Death (MedPed) Criteria [18]

**Age (years)**	**Total cholesterol cut points mmol/l (mg/dl)**			
	First-degree relative with FH	Second-degree relative with FH	Third-degree relative with FH	General population
< **20**	5.7 (220)	5.9 (230)	6.2 (240)	7 (270)
**20**–**39**	6.2 (240)	6.5 (250)	6.7 (260)	7.5 (290)
**30**–**39**	7 (270)	7.2 (280)	7.5 (290)	8.8 (340)
≥ **40**	7.5 (290)	7.8 (300)	8 (310)	9.3 (360)

## Lipid-Lowering Therapies for Hypercholesterolemia

Statins are still considered the first-line therapy for FH and can reduce LDLC by 50% from baseline. Statins block HMGCR and therefore inhibit HMG-CoA conversion to mevalonate, thereby reducing cholesterol synthesis [8, 10]. However, statins ‘ LDLC-lowering effect in FH patients is less stronger than in non-FH patients, and single therapy with statins is rarely sufficient to achieve the target LDLC level [10]. Therefore, combination with other lipid-lowering therapy is often necessary [26]. However, statins also lower LDLC through the sterol response element-binding protein 2 (SREBP2)/LDLR pathway. SREBP2 is a sensor that is sensitive to endoplasmic reticulum (ER) cholesterol content/concentration. As HMGCR inhibition reduces cholesterol synthesis, SREBP2 senses reduced ER cholesterol and upregulates LDLR by activating LDLR messenger-RNA (mRNA) transcription. Consequently, this leads to increased LDLR expression that increases LDLC uptake [12].

Ezetimibe is the second-line therapy for FH or first-line therapy in statin intolerance as recommended by the National Institute for Health and Care Excellence (NICE) [27]. Ezetimibe reduces cholesterol by blocking cholesterol absorption from the intestine through Niemann-Pick C1-like 1 protein (NPC1L1) blockage. NCP1L1 facilitates cholesterol absorption and internalizes cholesterol molecules. The NCP1L1/cholesterol complex then creates vesicles in the enterocyte after combining with other molecules called clathrin and adaptor protein 2 (AP2) [28]. In jejunal microvilli, ezetimibe also blocks enterohepatic cholesterol absorption. Hence, ezetimibe blocks both dietary and biliary cholesterol absorption [3]. Ezetimibe reduces LDLC by 18% and 25% when used as monotherapy and as a combination with statins, respectively [26].

Similar to statins, bempedoic acid (BA) blocks cholesterol synthesis. BA blocks acyl citrate lyase (ACL), the enzyme that cleaves citrate to synthesize acetyl-CoA. Therefore, blocking ACL reduces acetyl-CoA synthesis, which further reduces cholesterol synthesis [29]. BA was shown to reduce LDLC with a 17.4% difference when compared with placebo in FH patients [30]. Other less-commonly used drugs such as niacin (inhibits triglyceride synthesis that is essential for VLDL formation) and bile acid sequesterant (binds bile acids and prevents them from reaching enterohepactic circulation) were shown to reduce LDLC by 25% [10, 31, 32]. Importantly, in a study that included FH patients, a fixed-dose combination of BA and ezetimibe (FDA approved) has been shown to reduce LDLC by 36.2%, which was significantly lower than BA (17.2%) or ezetimibe (23.2%) alone [33].

A novel drug class, PCSK9 inhibitors, lowers LDLC by 50–65% in a dose-dependent manner [10]. PCSK9 inhibitor, as monoclonal antibodies (e.g., evolocumab, alirocumab), can reduce LDLR degradation by competitively binding with PCSK9 [34]. This prevents PCSK9 from binding with LDLR and therefore increases LDLC uptake due to increased LDLR availability. The PCSK9 inhibitor can also reduce LDLR degradation through gene silencing in the form of small interfering ribonucleic acid (siRNA) (e.g., inclisiran). Inclisiran binds with *PCSK9* mRNA, thereby preventing ribosomal translation and therefore inhibiting PCSK9 protein synthesis [35]. Inclisiran showed an almost 40% LDLC reduction in those optimally treated with statins (with or without ezetimibe) [36]. Several trials have robustly demonstrated PCSK9 inhibitor efficacy and safety in FH treatment [37–39].

A human recombinant monoclonal antibody, evinacumab, has been approved for homozygous monogenic FH or refractory hypercholesterolemia therapy. Evinacumab inhibits the activity of angioprotein-like protein three (ANGPTL3), thereby leading to preserved LPL and endothelial lipase activity. Thus, ANGPTL3 inhibition reduces LDLC regardless of LDLR function or availability on target cells [40]. Evinacumab, in a phase III clinical trial, was shown to reduce LDLC by 47.1% from baseline in optimally treated homozygous FH patients [41].

In addition to all the aforementioned therapies, some other therapies are in the pipeline. These include injectable antisense oligonucleotide (ASO) for PCSK9 (complementary to RNA sense strand that encodes *PCSK9*) that demonstrated a 43% LDLC reduction in an animal study [42, 43]. Furthermore, PCSK9 vaccine that stimulates host antibody production against PCSK9 and orally administered ASO might, in the future, overcome limitations stemming from the preceding therapies (e.g., repetitive administration due to short half-life, expensive, or invasive administration (injection)) [44, 45]. Moreover, a significant LDLC reduction was documented in a mice study following *LDLR* gene correction via the clustered regularly interspaced short palindromic repeat-CRISPR-associated protein 9 (CRISPR-Cas9) method [46].

It is worth noting that FH patients may also have elevated lipoprotein-a (Lp(a)), which has a similar molecular structure to LDLC and promotes atherosclerotic plaque and thrombus growth [47]. Thus, as demonstrated by Pavanello et al., FH patients with a history of previous cardiovascular diseases had a twofold higher Lp(a) level [48]. As a result, Lp(a)-targeting drugs have been developed with promising results in reducing Lp(a) levels up to 80–97% of baseline in a dose-dependent manner [49–51].

As there are various approved treatment options and promising drugs in the pipeline, the future challenge will arguably be formulating the best possible treatment for hypercholesterolemia patients. In accordance with that notion, patients’ genotype may provide crucial information that influences therapeutic decisions and patient outcomes.

## Cardiovascular Risk Profile Difference Between Monogenic and Polygenic Hypercholesterolemia

Trinder et al. discovered that monogenic FH compared to polygenic hypercholesterolemia had higher LDLC (7.08 mmol/l (273.8 mg/dl) vs. 5.72 mmol/l (221.2 mg/dl); *p* < 0.0001) and risk of premature (< 55 years old) CHD (hazard ratio 1.97 vs. 1.39), while polygenic hypercholesterolemia had similar risk with hypercholesterolemia without identified LDLC-raising variants [6]. Khera et al. demonstrated that among all patients whose LDLC was 190–220 mg/dl, the risk of CHD increased 17-fold in those with identified monogenic mutations, while in those without increased fivefold. This significant difference was observed despite both groups having similar mean LDLC (205 mg/dl in those with mutation vs. 203 mg/dl in those without) [52]. Furthermore, those with monogenic mutations and LDLC > 190 mg/dl had a 22-fold higher risk for CHD, while those with LDLC > 190 mg/dl without monogenic mutation had a sixfold increase compared to the reference group (LDLC < 130 mg/dl and without mutation). Monogenic mutation carriers also had a lifetime higher risk of CHD than non-carriers at any given LDLC and were associated with a 3.8-fold and 50 mg/dl increase of CHD risk and LDLC level, respectively [52]. However, this study did not determine a polygenic group as the comparison for monogenic FH since no polygenic score calculation was made. Nevertheless, this study confirmed that monogenic hypercholesterolemia had a higher risk of CAD. Similarly, Trinder et al. demonstrated that those with monogenic FH were more likely to experience CAD at ≤ 55 years old than those without [6].

A study involving 86 FH patients (56 monogenic vs. 30 polygenic) measured carotid intima-medial thickness (IMT) using B-mode ultrasound. This study also measured coronary artery calcium (CAC) using CT scan on 166 FH patients (124 monogenic vs. 42 polygenic). Carotid IMT in monogenic patients was higher than in polygenic (0.74 mm vs. 0.66 mm, *p* 0.038). A CAC score (reported in Agatson unit) of < 100 was generally shown to have very low ischemia prevalence, and therefore, 100 was used as the cutoff. Monogenic FH patients had a higher prevalence and odds ratio of having a CAC score > 100 compared to the polygenic group (prevalence 41% vs. 28.6%; odds 4.79, *p* 0.004). Furthermore, CAC was 9.75 times higher (*p* 0.0004) in the monogenic compared to the polygenic group. Hence, monogenic FH patients tended to develop more severe atherosclerosis [19]. Similarly, D’Erasmo et al. also demonstrated that the monogenic group had a significantly higher mean CAC, a CAC score > 100, and a greater number of coronary plaques than polygenic group [2•].

## Different Response of Monogenic and Polygenic Hypercholesterolemia on Treatment

Several studies have shown that genetic background influences the therapeutic response on FH patients. For instance, patients with an *LDLR* null mutation showed the poorest response than those with defective and without mutations on atorvastatin therapy. Among all patients who attained an after-treatment LDLC level of < 3.4 mmol/l (± 130 mg/dl), only 22.5% were patients with null mutations, while the defective and no-mutation groups were 27.1% and 47.4%, respectively (*p* 0.02). Furthermore, those with *LDLR* mutations were 9.07 times less likely to achieve targeted LDLC than those without [21].

Mickiewicz et al., demonstrated a higher reduction in LDLC in the polygenic group compared to the monogenic FH (55.4% vs. 45.9%; *p* < 0.001) after adjusting for covariates (different rosuvastatin dose, ezetemibe use, and LDLC level at baseline) upon rosuvastatin therapy. The study also demonstrated that polygenic hypercholesterolemia had a higher probability of achieving LDLC target than monogenic FH (24.5% vs. 7.5%; *p* 0.004). Furthermore, the polygenic patients were also 3.28 times more likely to achieve the targeted LDLC level than monogenic FH [4•]. Moreover, a study on 370 clinically diagnosed FH patients by D’Erasmo et al. also yielded similar conclusions. The patients were classified as monogenic, polygenic, and undetermined genetic causes based on Sanger sequencing and PRS calculation. Monogenic FH patients showed the poorest response to lipid-lowering therapy of different regiments and intensity, while the other groups were comparable. Monogenic patients were also at a 5 times higher risk of experiencing atherosclerotic cardiovascular events than other groups [2•].

On the contrary, Lee et al. studied 39 patients (32 monogenic heterozygous FH vs. 7 polygenic hypercholesterolemia). The study concluded that there was no significant proportion difference in both groups who achieved the LDLC target of > 50% reduction on evolocumab therapy [53]. Nevertheless, despite no significant difference observed, the mean absolute and percentage of LDLC reduction were slightly higher in polygenic than in monogenic hypercholesterolemia (3.15 mmol/l (121.8 mg/dl) vs. 2.94 mmol (113.7 mg/dl); 67.7% vs. 63.9%). However, this study was conducted with a small sample size and a large discrepancy in the proportion of studied groups, which might not be robust enough to find a significant difference.

## Clinical Implications

Because monogenic FH has a higher cardiovascular risk independent of LDLC level and is less responsive to therapy than polygenic FH [2•, 6, 52], distinguishing between the two is important for risk stratification and therapy. Thus, genetic testing should be performed whenever possible. This is also a good practice of precision medicine [17]. Genetic testing can also assist early diagnosis, as clinical diagnosis alone misses up to 80% of FH patients, especially those without or mild lipid and clinical presentation and no family history of premature CHD [11]. This is especially essential in the pediatric population since the LDLC threshold for diagnosis is generally lower [11]. Furthermore, as FH diagnosis and epidemiological data are primarily based on patients who survived myocardial infarct [25], identifying mutations/variants before clinical and lipid abnormalities manifest is essential to reduce mortality or morbidity from early CHD.

As monogenic FH is less likely to achieve the target LDLC value, obtaining genotype information is essential in deciding drugs of choice or when to deliver more aggressive therapy [4•, 11]. Moreover, LDLC value alone is not sufficient to determine prognosis as similar LDLC value may have significantly different CAD risk when the genotype is different [52]. Hence, genotype-guided therapy is more relevant than LDLC level-guided therapy for risk stratification and prognostication. A study on 622 possible FH patients diagnosed with SBRG criteria demonstrated that genetic diagnosis in FH is superior to clinical diagnosis as it allows for early case recognition, stratification, and treatment [54], while LDLC-based diagnosis alone results in up to 18% of false negative and false positive cases [25]. Therefore, there is an urgent need to develop genotype-based clinical guidelines and to consider genotype in stratifying hypercholesterolemia patients rather than stratifying patients based on the conventional cardiovascular risks alone. In the meantime, the best option is arguably individualizing therapy for FH and polygenic hypercholesterolemia with respect to LDLC level, clinical presentation and history, and other cardiovascular risk factors.

Furthermore, knowing a patient’s genotype can also affect disease detection and screening. In polygenic hypercholesterolemia, cascade testing is less efficient as only 30% of relatives have elevated LDLC compared to 50% in monogenic FH [5••]. However, some areas require further study, including the necessity of distinguishing genotype in PCSK9 inhibitor therapy, as a study demonstrated a comparable result upon evolocumab therapy in both monogenic and polygenic groups [53]. This is in accordance with D’Erasmo et al. who also indicated that PCSK9 inhibitor effect is independent of the genotype [2•].

## Conclusion

Monogenic FH has a worse cardiovascular risk profile and responsiveness to lipid-lowering therapy compared to polygenic hypercholesterolemia. Therefore, whenever possible, genetic diagnosis should be performed to assist with clinical diagnosis. Identifying genetic background (monogenic or polygenic) can reinforce early diagnosis, determine drug of choice and intensity, disease prognosis, and optimize disease screening. Moreover, there is an urgency to develop separated therapeutic pathway for both disease entities and to consider genotype in patient stratification. Further investigation is also required to robustly determine whether genotype differentiation is necessary for PCSK9-inhibitor treatment.
